# Editorial: Theoretical study of two-dimensional materials for photocatalysis and photovoltaics

**DOI:** 10.3389/fchem.2024.1387236

**Published:** 2024-03-06

**Authors:** Kai Ren, Jefferson Zhe Liu, Maurizia Palummo, Minglei Sun

**Affiliations:** ^1^ School of Mechanical and Electronic Engineering, Nanjing Forestry University, Nanjing, China; ^2^ Department of Mechanical Engineering, The University of Melbourne, Parkville, VIC, Australia; ^3^ Dipartimento di Fisica and INFN, Università di Roma ‟Tor Vergata”, Roma, Italy; ^4^ Department of Physics and NANOlab Center of Excellence, University of Antwerp, Antwerp, Belgium

**Keywords:** two-dimensional, heterostructure, defect, photocatalysis, photovoltaics

## Introduction

To alleviate the global energy shortage and address environmental pollution, hydrogen (H_2_) is being considered as a promising clean energy source because its combustion only generates water (Kud et al., 2009). Currently, despite the abundant solar energy offered by sunlight, its conversion efficiency is generally low, showing the necessity of using sunlight to cataluze the decomposition of water into H_2_. Compared with bulk materials, using two-dimensional (2D) materials as a photocatalyst for water splitting is more advantageous, because 2D photocatalysts have a larger specific surface area, which can provide more catalytic activity sites for redox reactions (Zhang et al., 2023a). In addition, the recombination of photogenerated electrons and holes on the surface of the catalyst presents a real challenge for redox reactions. The introduction of 2D type-II heterostructures addresses this issue by separating the photogenerated electrons and holes across different layers, thereby enhancing the longevity of the photogenerated charges. Therefore, exploring 2D materials for photocatalytic water splitting is of critical (Ren et al., 2020) importance. To date, a large number of 2D materials have been suggested or synthesized, including graphene (Geim and Novoselov, 2007), molybdenum disulfide (MoS_2_) (Mak et al., 2010), blue phosphorus (Gu et al., 2017), and arsenene (Zhang et al., 2015). These materials are distinguished by their unique physical and chemical properties, making them highly suitable for a wide range of applications in optoelectronics, thermoelectrics, photovoltaics, and catalysis. Furthermore, novel 2D material prediction (Ren et al., 2022a), strain engineering (Li et al., 2023), adsorption (Ren et al., 2022b), doping (Chen et al., 2024), defect (Luo et al., 2023), size effects (Ren et al., 2023), and the application of external electric fields (Sun et al., 2017) have proven to be effective approaches to further expand the applications of 2D materials.

In parallel, computer hardware is developing rapidly, and numerical calculation methods are also constantly being improved, yielding more efficient, reliable, and accurate methods. Among them, the first-principles calculation method, based on density functional theory (DFT) (Kresse and Furthmüller, 1996), is widely used to investigate various properties of nanomaterials. Moreover, the first-principles calculation method shows results that closely align with the experimental values (Singh et al., 2015). It is also able to predict the properties of 2D materials, offering a theoretical foundation for experimental efforts and exploring their potential applications (Zhuang and Hennig, 2013).

In this Research Topic “*Theoretical Study of Two-Dimensional Materials for Photocatalysis and Photovoltaics*,” we have collected a total of seven articles, including the recent study on tuning the properties of 2D materials using the theoretical calculation method. We will now briefly summarize the research highlights in these fascinating articles.

2D ferroelectric heterostructures are constructed based on C_2_N and α-In_2_Se_3_ layers in the paper by Zhong. The optoelectronic property of the C_2_N/α-In_2_Se_3_ heterostructure with varied polarization orientations is addressed by first-principal simulations. Interestingly, the traditional type-II heterostructure with an indirect bandgap of 0.63 eV can be transformed to an S-scheme heterostructure by the ferroelectric polarization of α-In_2_Se_3_ reversed from up to down. The work function and the charge density difference demonstrate that the C_2_N/α-In_2_Se_3_ S-scheme heterostructure is a promising photocatalyst.

Zhang and Cui constructed nine different non-metal-doped silicon carbide (NM-SiC) systems and then addressed the magnetic, electronic, and optical performances systematically based on density functional theory (DFT). The most stable structure of the NM-SiC was decided by the maximum binding energy. Furthermore, the O-, Si-, and S-SiC configurations were investigated as non-magnetic semiconductors, while the N- and P-SiC configurations present magnetic behavior as semiconductors. In addition, the H-, F-, and Cl-SiC configurations showed a half-metal characteristic, and the B-SiC system acted as a magnetic metal. The doping of NM atoms is a popular measure to tune the work function of the NM-SiC systems, as this can obtain the minimal work function of 3.70 eV in the P-SiC, which is 77.1% of the SiC. The absorption spectrum of the NM-SiC layers also presented red-shift in the ultraviolet light part, with the absorption coefficient decreasing. The results explain the potential application of spintronics devices and designing field emission with NM-SiC systems.

In their article, Shen et al. selected 2D ZnO as an adsorbed substrate with M (Li, Na, Mg, Ca, or Ga) and TM (Cr, Co, Cu, Ag, or Au) atoms, and the electronic structures, magnetic properties, and optical performances were systematically explored by first-principle calculations based on DFT. The band structure, charge density difference, electron spin density, work function, and absorption spectrum of ZnO obviously can be tuned by adsorbing M or TM atoms. In particular, the decent charge transfer in ZnO and adsorbed atom shows the formation of a covalent bond. Summarily, the work function of the M-adsorbed ZnO structure is significantly smaller than the intrinsic ZnO monolayer, suggesting it to be a promising candidate as a high-efficiency field emission device. The Li-, Na-, Mg-, Ca-, Ga-, Ag-, and Au-adsorbed ZnO structure presents a magnetic semiconductor property, while the Cr-adsorbed ZnO systems are non-magnetic semiconductors. The Co- and Cu-adsorbed ZnO systems also demonstrated magnetic metal characteristic. Furthermore, the magnetic moment of the Cr-, Co-, and Cu-adsorbed ZnO systems were obtained as 4 *μ*_B_, 3 *μ*_B_, and 1 *μ*_B_, respectively, which are mainly contributed to by adsorbed atoms, showing their potential for use in nano-scale spintronics devices. The M-adsorbed ZnO configurations show more apparent absorption peaks in visible light than the TM-absorbed ZnO systems, particularly for Mg- or Ca-adsorbed ZnO systems. Importantly, the calculated absorption peaks in the near-infrared region suggest potential applications in solar photocatalysis. This work provides theoretical guidance for the design and fabrication of high-efficiency field emission devices, visible-light photocatalysts, and spintronics devices.

Zhang and Cui. Further studied the electronic and optical performances of the monolayered blue phosphorene (BlueP) by the external strain from −10% to +10% using the DFT method. All the strained BlueP presented as stable, so they could induce a transformation from a metallic to direct semiconductor from −10% to 10% strain. Such a phenomenon resulted from the competition of the energy states near the Fermi level under a massive strain. The decent compressive strain can cause the *p*_*y*_ orbitals of the conduction band to move downward and pass the Fermi level at the K point. The strong tensile strain guides the energy state of the Γ point close to the Fermi level and becomes the band edge. At the same time, the strained BlueP is still an indirect semiconductor under the strain of −8% to +8%. Even if the bandgap of the BlueP is overall linearly changed by strain, the bandgap of the BlueP possesses a stronger dependence on the tensile strain compared to the compressive strain. Meanwhile, the real part of the dielectric function of BlueP can be evidently improved by the compressive strain. The maximal absorption coefficient of the BlueP was obtained as 0.52 × 10^5^/cm with the wavelength at 530 nm under the strain as 10%. Their investigation suggests a practical application for BlueP in electronic devices, photovoltaic cells, and photocatalysts.

Monoelemental 2D materials have attracted abundant development interest due to their fascinating performances. Zhang et al. investigated the phonon transport and thermoelectric properties of tellurium at different layers with first-principles calculations. They found that the anisotropy of the thermal transport characteristic of tellurium is suppressed by the increased layers. The enhanced phonon transport by the layer is decided by increasing the phonon velocity in specific phonon modes using the phonon-level systematic method. The thermoelectric transport performance presented a maximal figure of merit of about 6.3 in armchair direction at 700 K in monolayered tellurium, while presenting 6.6 (p-type) in the zigzag direction in bilayer tellurium at 700 K, suggesting apparent anisotropic thermoelectric behavior. This work shows tellurium has tremendous potential for use in thermoelectric applications.

Considering the global energy crisis, hydrogen has the advantage of high combustion and shows considerable environmental friendliness; however, the main obstacle to fully utilizing this new resource lies in its transportation and storage. Chen et al. investigated the 2D g-C_3_N_5_ with hydrogen as a storage material. First-principles calculations are conducted so that the charge of the added Li atom can be transferred from g-C_3_N_5_ to the adjacent nitrogen atom, forming a chemical interaction. Therefore, the isolated metal sites often exhibit considerable electropositivity and can easily polarize adsorbed hydrogen molecules, thus, the electrostatic interactions can also be improved accordingly. Each original cell has a maximal storage capacity of up to 20 hydrogen molecules, with a gravimetric capacity of 8.65 wt%, exceeding the 5.5 wt% target set by the U.S. Department of Energy. The obtained average adsorption energies range from −0.22 to −0.13 eV. Their study concludes that the complex Li-decorated g-C_3_N_5_ can serve as a promising hydrogen storage medium. Wang et al. also focus on energy storage strategies and designed a set of Tesla turbines. The silicon steel sheet material is selected for the rotor as it possesses obviously excellent rotor dynamics and flow field characteristics, which provide new ideas for boundary layer effects.

We hope that this Research Topic can provide guidance for developing novel 2D photocatalysis and photovoltaics. We thank all the authors, reviewers, and editors who have made contributions to this Research Topic.
